# Phenotypic variability in patients with ADA2 deficiency due to identical homozygous R169Q mutations

**DOI:** 10.1186/1546-0096-13-S1-O7

**Published:** 2015-09-28

**Authors:** J Van Montfrans, E Hartman, K Braun, F Hennekam, A Hak, P Nederkoorn, W Westendorp, R Bredius, W Kollen, E Scholvinck, G Legger, I Meyts, A Liston, K Lichtenbelt, J Giltay, G Van Haaften, G De Vries Simons, H Leavis, S Nierkens, C Sanders, M Van Gijn

**Affiliations:** 1 University Medical Center Utrecht, Pediatric Immunology and Infectious Diseases, Utrecht, Netherlands; 2 University Medical Center Utrecht, Child Neurology, Utrecht, Netherlands; 3 University Medical Center Utrecht, Medical Genetics, Utrecht, Netherlands; 4 Academic Medical Center, Amsterdam Rheumatology and immunology Center, Amsterdam, Netherlands; 5 Academic Medical Center, Neurology, Amsterdam, Netherlands; 6 Leiden University Medical Center, Pediatrics, Leiden, Netherlands; 7 University Medical Center Groningen, Paediatrics, Groningen, Netherlands; 8 University Hospital Leuven, Paediatrics, Leuven, Belgium; 9 University Hospital Leuven, Microbiology and Immunology, Leuven, Belgium; 10 University Medical Center Utrecht, Rheumatology and Clinical Immunology, Utrecht, Netherlands; 11 University Medical Center Utrecht, U-DAIR, Laboratory of Translational Immunology, Utrecht, Netherlands

## Introduction

Deficiency of adenosine deaminase-2 (ADA2) is a recently described autoinflammatory disorder with cutaneous inflammatory disease, febrile episodes, cytopenias, splenomegaly and early-onset stroke. Several homozygous and compound heterozygous mutations in *CECR1* have been reported in these patients; however, pathogenesis is still poorly understood.

## Objective

To determine the genotype - phenotype association in patients with ADA2 deficiency due to identical homozygous R169Q mutations in CECR1.

## Methods

We performed a cohort study in nine patients diagnosed with ADA2 deficiency due to a homozygous R169Q mutation in the Netherlands and Belgium. Clinical and diagnostic data were collected from clinical files. We performed genealogy and haplotype analyses and measured serum ADA2 activity. ADA2 activity values were correlated to clinical symptoms.

## Results

Age of presentation differed widely between patients (range: 0 mths to 8 yrs). The main clinical manifestations were (hepato)splenomegaly (9/9); skin involvement (8/9) and neurological involvement (8/9, of whom 6 encountered stroke). Considerable variation was seen in type, frequency and intensity of other symptoms, which included aplastic anemia, acute myeloid leukemia and cutaneous ulcers. Common laboratory abnormalities included cytopenias and hypogammaglobulinemia. ADA2 enzyme activity in patients was significantly decreased compared to healthy controls (0.78 vs 5.41 IU/L, p < 0.0001). Within the patient cohort, ADA2 activity levels tended to be lower in patients with stroke compared to patients without stroke (0.30 vs 1.57 IU/L, p= 0.064). No common ancestor for all families could be detected by genealogy, however, based on allele frequency, a Dutch founder effect can be noted. Three patients underwent hematopoietic cell transplantation, after which ADA2 activity was restored and clinical symptoms resolved.

## Conclusions

This study revealed large phenotypic variability in patients with ADA2 deficiency though they carried the same homozygous R169Q mutation in CECR1. Epigenetic and environmental factors thus seem important in the phenotype. A trend towards a relation between stroke risk and low ADA2 residual activity was seen. Furthermore, hematopoietic stem cell transplantation appears promising for those patients with a severe clinical phenotype.

